# Molluscan fauna of Gueishan Island, Taiwan

**DOI:** 10.3897/zookeys.261.4197

**Published:** 2013-01-24

**Authors:** Chih-Wei Huang, Ta-Wei Hsiung, Si-Min Lin, Wen-Lung Wu

**Affiliations:** 1 Department of Life Science, National Taiwan Normal University, No. 88, Sec. 4, Tingzhou Rd., Wenshan Dist., 11677, Taipei, TAIWAN, R.O.C.; 2 Biodiversity Research Center, Academia Sinica, No. 128 Academia Road Sec. 2, Nankang Dist., 11529, Taipei, TAIWAN, R.O.C.

**Keywords:** Mollusca, Gastropoda, Bivalvia, Cephalopoda, Polyplacophora, Taiwan, Gueishan Island

## Abstract

This dataset records the occurrence and inventory of molluscan fauna on Gueishan Island, the only active volcanic island in Taiwan, based on the literature survey and field investigation conducted between 2011 and 2012. The literature review involved seven studies published from 1934 to 2003, which collectively reported 112 species from 61 genera and 37 families of Mollusca on Gueishan Island. Through our field investigation, we identified 34 species from 28 genera and 23 families. Fourteen of these species were new records on Gueishan Island: *Liolophura japonica*, *Lottia luchuana*, *Nerita costata*, *Nerita rumphii*, *Diplommatina suganikeiensis*, *Littoraria undulata*, *Solenomphala taiwanensis*, *Assiminea* sp., *Siphonaria laciniosa*, *Laevapex nipponica*, *Carychium hachijoensis*, *Succinea erythrophana*, *Zaptyx crassilamellata*, and *Allopeas pyrgula*. In Total, there are 126 species from 71 genera and 45 families of Mollusca on Gueishan Island. These data have been published through GBIF [http://taibif.org.tw/ipt/resource.do?r=gueishan_island] and integrated into the Taiwan Malacofauna Database (http://shell.sinica.edu.tw/).

## Project details

**Project title:** Investigation of molluscan fauna of Gueishan Island, Taiwan.

**Personnel:** Chih-Wei Huang (collection identifier, data collector, data manager, data publisher), Ta-Wei Hsiung (collection identifier, data collector, data manager), Yen-Chen Lee (collection identifier), Si-Min Lin (Project Director), Wen-Lung Wu (Project Director, data manager).

**Funding:** Academia Sinica; National Science Council, Executive Yuan, R.O.C.(Taiwan); Forest Bureau, Council of Agriculture, Executive Yuan, R.O.C.(Taiwan).

**Figure 1.**
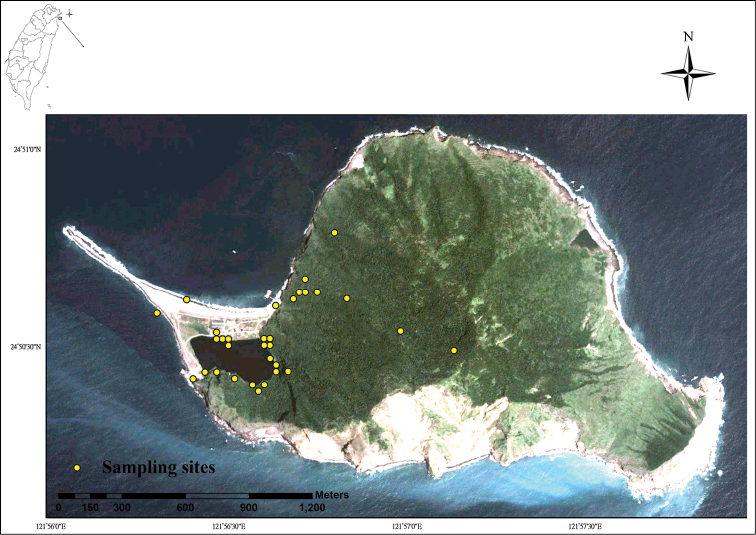
Location of Gueishan Island and field sampling sites of this study.

**Study area descriptions/descriptor:** Gueishan Island is located about 10 km from Taiwan. The island was formed via volcanic activity about 1.65 Ma ago and experienced multiple volcanic eruption events until 20 ka ago (Juang et al. 2011). It is considered the only active volcanic island near Taiwan. The land area of the island is about 2.85 km^2^, and the highest peak of the island is 398 meters above sea level. There are two lakes on the island, one of which consist of brackish water (Head Lake) and the other of freshwater (Tail Lake) (Figure 1). Humans colonized Gueishan Island in mid-19th century, by forming a small village. Later in 1977, all residents were moved back to Taiwan due to military requirements for the island. The fauna of this island were not investigated systematically until 2000, when the island came under the management of the Northeast and Yilan Coast National Scenic Area Administration, Tourism Bureau, MOTC and was open to tourists.

**Design description:** Island species are vulnerable to extinction due to their relatively small population size and limited access to resources. The number of species on an island represents a dynamic equilibrium between immigration and extinction. Volcanic islands provide particularly interesting cases of island biogeography, in that their biota is erased by volcanic activity and recolonized from neighboring regions. Species on Gueishan Island may have under gone several cycles of extinction after volcanic eruption, followed by recolonization from Taiwan when the sea-level dropped during glacial periods. Human activity may also have provided opportunities for colonization of mollusks, either intentionally or accidentally. Investigations of molluscan fauna have been previously conducted on Gueishan Island, but these did not involve a comprehensive examination of land snails. We performed a literature survey using diverse databases, in order to collect previously identified reports on molluscan fauna of Gueishan Island. In addition, we performed field sampling of mollusks in marine, freshwater and terrestrial environments during 2011 and 2012 to establish the inventory of molluscan fauna of Gueishan Island. We considered both the topography of the island and the habitats of mollusks during our field investigation. We focused on the terrestrial environment, as the majority of the earlier investigations examined non-terrestrial habitats. In total, our literature survey and field investigation identified 126 species from 71 genera and 45 families of Mollusca on Gueishan Island. This dataset provides basic information on the island’s biodiversity.

## Taxonomic coverage

**Figure 2.**
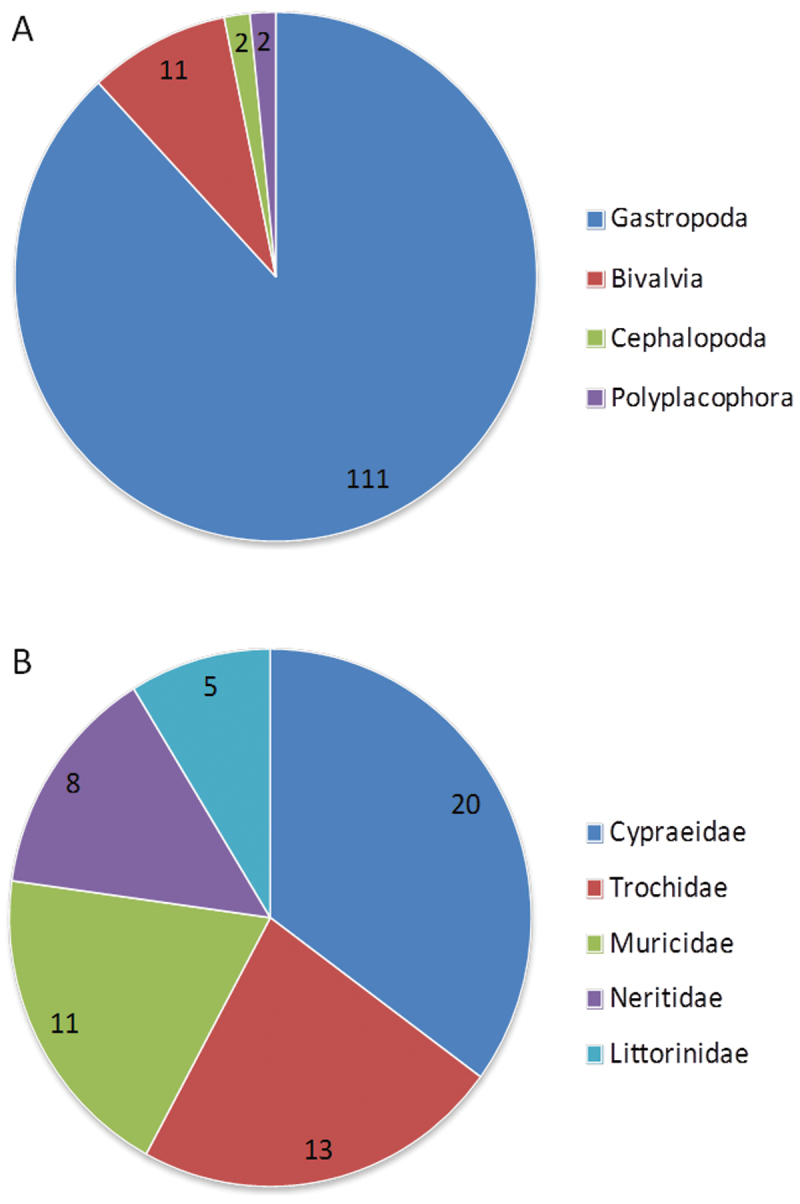
Taxonomic coverage. **A** Class **B** The top five representative families. Numbers in pie charts represent number of species.

**General taxonomic coverage description:** The coverage of this dataset includes 126 species from 71 genera and 45 families of Mollusks of marine, freshwater and terrestrial environments on Gueishan Island (Table 1). It includes Class Gastropoda (88.10%), Class Bivalvia (8.73%), Class Cephalopoda (1.59%), and Class Polyplacophora (1.59%). The top five representative families are Cypraeidae (20 species, 15.87%), Trochidae (13 species, 10.32%), Muricidae (11 species, 8.73%), Neritidae (8 species, 6.35%), and Littorinidae (5 species, 3.97%) (Figure 2).

**Table 1.** Species inventory of mollusks of Gueishan Island, Taiwan.

**Taxa**	**References**
CLASS POLYPLACOPHORA	
ORDER NEOLORICATA	
FAMILY CHITONIDAE	
†*Liolophura japonica* (Lischke, 1873)	§
*Liolophura* sp.	(Hwang and Lee 2003)
CLASS CEPHALOPODA	
ORDER OCTOPODA	
FAMILY ARGONAUTIDAE	
*Argonauta hians* (Lightfoot, 1786)	(Wu 2002)
*Octopus* sp.	(Hwang and Lee 2003)
CLASS BIVALVIA	
ORDER VENEROIDA	
FAMILY CORBICULIDAE	
*Corbicula fluminea* (Müller, 1774)	§ (National Museum of Marine Biology and Aquarium 2003)
FAMILY CARDIIDAE	
*Tridacna crocea* Lamarck, 1819	(Hwang and Lee 2003)
*Tridacna gigas* (Linnaeus, 1758)	(Hwang and Lee 2003)
*Tridacna maxima* (Roeding, 1798)	(Jung and Lai 1999)
ORDER UNIONOIDA	
FAMILY UNIONIDAE	
*Cristaria discoidea* (Lea, 1834)	(Hayasaka and Tan 1934)
ORDER ARCOIDA	
FAMILY ARCIDAE	
*Barbatia foliate* (Forskal, 1775)	(Hwang and Lee 2003)
ORDER OSTREOIDA	
FAMILY OSTREIDAE	
*Crassostrea gigas* (Thunberg, 1793)	(National Museum of Marine Biology and Aquarium 2003)
*Saccostrea mordax* (Gould, 1850)	(National Museum of Marine Biology and Aquarium 2003, Hwang and Lee 2003)
FAMILY PECTINIDAE	
*Chlamys irregularis* (Sowerby, 1842)	(Jung and Lai 1999)
ORDER PTERIOIDA	
FAMILY PTERIIDAE	
*Pinctada margaritifera* (Linnaeus, 1758)	(Jung and Lai 1999), Hwang and Lee 2003
*Pteria penguin* (Roeding, 1798)	Hwang and Lee 2003
CLASS GASTROPODA	
ORDER PATELLOGASTROPODA	
FAMILY PATELLIDAE	
*Cellana grata* (Gould, 1859)	§ (Jung and Lai 1999)
*Cellana toreuma toreuma* (Reeve, 1854)	§ (Jung and Lai 1999, Hwang and Lee 2003)
FAMILY LOTTIIDAE	
*Collisella heroldi heroldi* (Dunker, 1861)	(Hwang and Lee 2003)
†*Lottia luchuana* (Pilsbry, 1901)	§
*Notoacmea schrenckii schrenckii* (Lischke, 1868)	(Jung and Lai 1999, Wu 2002, Hwang and Lee 2003)
ORDER VETIGASTROPODA	
FAMILY HALIOTIDAE	
*Haliotis diversicolor* (Reeve, 1846)	(Jung and Lai 1999)
FAMILY TROCHIDAE	
*Calliostoma unicum* (Dunker, 1860)	(Jung and Lai 1999)
*Chlorostoma turbinatum* A.Adams, 1853	(Jung and Lai 1999)
*Chlorostoma argyrostoma argyrostoma* (Gmelin, 1791)	(Jung and Lai 1999, Wu 2002, Hwang and Lee 2003)
*Monodonta labio* (Linnaeus, 1758)	(Jung and Lai 1999, Hwang and Lee 2003)
*Monodonta perplexa* Pilsbry, 1889	§ (Jung and Lai 1999, Hwang and Lee 2003)
*Stomatella planulata* (Lamarck, 1816)	(Jung and Lai 1999)
*Tectus conus* (Gmelin, 1791)	(Jung and Lai 1999)
*Tectus pyramis* (Born, 1778)	(Jung and Lai 1999)
*Trochus chloromphalus* A. Adams, 1853	(Jung and Lai 1999)
*Trochus hanleyanus* Reeve, 1842	(Jung and Lai 1999, Wu 2002, Hwang and Lee 2003)
*Trochus maculates* Linnaeus, 1758	(Jung and Lai 1999, Hwang and Lee 2003)
*Trochus sacellum* Philippi, 1854	(Jung and Lai 1999, Hwang and Lee 2003)
*Trochus stellatus* Gmelin, 1790	(Jung and Lai 1999, Hwang and Lee 2003)
FAMILY TURBINIDAE	
*Astralium haematragum* (Menke, 1829)	(Hwang and Lee 2003)
*Lunella coronate* (Gmelin, 1818)	(Hwang and Lee 2003)
ORDER NERITIMORPHA	
FAMILY NERITIDAE	
*Nerita albicilla* Linnaeus, 1758	(Jung and Lai 1999, Hwang and Lee 2003)
†*Nerita costata* Gmelin, 1791	§
*Nerita plicata* Linnaeus, 1758	§ (Jung and Lai 1999)
†*Nerita rumphii* Recluz,1841	§
*Nerita chamaeleon* Linnaeus, 1758	(Hwang and Lee 2003)
*Nerita ocellata* Leguillou, 1841	(Jung and Lai 1999)
*Nerita polita* Linnaeus, 1758	(Jung and Lai 1999)
*Nerita undata* Linnaeus, 1758	(Jung and Lai 1999)
ORDER CAENOGASTROPODA	
FAMILY DIPLOMMATINIDAE	
†*Diplommatina suganikeiensis* (Pilsbry & Hirase, 1905)	§
FAMILY PLANAXIDAE	
*Planaxis sulcatus* (Born, 1778)	(Hwang and Lee 2003)
FAMILY POTAMIDIDAE	
*Batillaria zonalis* (Bruguiere, 1792)	(National Museum of Marine Biology and Aquarium 2003)
FAMILY THIARIDAE	
*Tarebia granifera* (Lamarck, 1822)	§ (National Museum of Marine Biology and Aquarium 2003)
*Thiara scabra* (Muller, 1774)	§ (National Museum of Marine Biology and Aquarium 2003)
*Thiara tuberculata* (Muller 1774)	§ (National Museum of Marine Biology and Aquarium 2003)
FAMILY CYPRAEIDAE	
*Cypraea annulus* Linnaeus, 1758	(Jung and Lai 1999, Hwang and Lee 2003)
*Cypraea arabica* Linnaeus, 1758	(Jung and Lai 1999)
*Cypraea asellus* Linnaeus, 1758	(Jung and Lai 1999)
*Cypraea caputserpentis* Linnaeus, 1758	(Jung and Lai 1999, Hwang and Lee 2003)
*Cypraea caurica* Linnaeus, 1758	(Jung and Lai 1999)
*Cypraea chinensis* Gmelin, 1791	(Jung and Lai 1999)
*Cypraea clandestine* Linnaeus, 1758	(Jung and Lai 1999)
*Cypraea eglantine* Duclos, 1833	(Hwang and Lee 2003)
*Cypraea erosa* Linnaeus, 1758	(Jung and Lai 1999)
*Cypraea gracilis* Gaskoin, 1849	(Jung and Lai 1999)
*Cypraea helvola* Linnaeus, 1758	(Jung and Lai 1999)
*Cypraea labrolineata* Gaskoin, 1849	(Jung and Lai 1999)
*Cypraea lynx* Linnaeus, 1758	(Hwang and Lee 2003)
*Cypraea moneta* Linnaeus, 1758	§ (Jung and Lai 1999, Hwang and Lee 2003)
*Cypraea onyx* Linnaeus, 1758	(Jung and Lai 1999)
*Cypraea poraria* Linnaeus, 1758	(Jung and Lai 1999)
*Cypraea testudinaria* Linnaeus, 1758	(Jung and Lai 1999)
*Cypraea tigris* Linnaeus, 1758	(Jung and Lai 1999)
*Cypraea ziczac* Linnaeus, 1758	(Jung and Lai 1999)
*Cypraea errones* Linnaeus, 1758	(Wu 2002)
FAMILY OVULIDAE	
*Calpurnus verrucosus* (Linnaeus, 1758)	(Hwang and Lee 2003)
*Ovula ovum* Linnaeus, 1758	(Hwang and Lee 2003)
FAMILY LITTORINIDAE	
*Littoraria pintado* (Wood, 1828)	(Jung and Lai 1999)
†*Littoraria undulate* (Gray, 1839)	§
*Littoraria scabra scabra* (Linnaeus, 1758)	(Hwang and Lee 2003)
*Nodilittorina pyramidalis* (Quay & Gaimard, 1833)	§ (Jung and Lai 1999, Hwang and Lee 2003)
*Nodilittorina vidua* (Gould, 1859)	§ (Jung and Lai 1999, Hwang and Lee 2003)
FAMILY ASSIMINEIDAE	
†*Solenomphala taiwanensis* (Habe, 1942)	§
†*Assiminea* sp.	§
FAMILY BURSIDAE	
*Bursa granularis* (Roeding, 1798)	(Jung and Lai 1999)
FAMILY RANELLIDAE	
*Cymatium aquatile* (Reeve, 1844)	§ (Jung and Lai 1999)
*Cymatium mundum* (Gould, 1849)	(Jung and Lai 1999)
*Cymatium pileare* (Linnaeus, 1758)	(Jung and Lai 1999)
*Cymatium lotorium* (Linnaeus, 1758)	(Hwang and Lee 2003)
FAMILY COLUMBELLIDAE	
*Pyrene punctata* (Bruguiere, 1789)	(Jung and Lai 1999)
*Pyrene testudinaria testudinaria* (Link, 1806)	(Hwang and Lee 2003)
FAMILY FASCIOLARIIDAE	
*Peristernia nassatula* (Lamarck, 1822)	(Wu 2002)
FAMILY NASSARIIDAE	
*Nassarius glans* (Linnaeus, 1758)	(Jung and Lai 1999)
*Nassarius papillosus* (Linnaeus, 1758)	(Jung and Lai 1999)
*Telasco velatus* (Gould, 1850)	(Jung and Lai 1999)
FAMILY MURICIDAE	
*Chicoreus torrefactus* Sowerby, 1841	(Wu 2002)
*Chicoreus brunneus* (Link, 1807)	(Hwang and Lee 2003)
*Drupa morum* Roeding, 1798	(Jung and Lai 1999)
*Drupa ricina ricina* (Linnaeus, 1758)	(Jung and Lai 1999, Wu 2002, Hwang and Lee 2003)
*Drupa rubusidaea* Roeding, 1798	(Jung and Lai 1999, Hwang and Lee 2003)
*Ergalatax contractus* (Reeve, 1846)	(Jung and Lai 1999, Hwang and Lee 2003)
*Mancinella mancinella* (Linnaeus, 1758)	§ (Jung and Lai 1999)
*Morula uva* (Roeding, 1798)	(Wu 2002)
*Purpura panama* (Roeding, 1798)	(Jung and Lai 1999, Wu 2002)
*Tenguella granulate* (Duclos, 1924)	§, (Jung and Lai 1999)
*Thais clavigera* (Kuster, 1860)	(Wu 2002, Hwang and Lee 2003)
FAMILY TURBINELLIDAE	
*Vasum ceramicum* (Linnaeus, 1758)	(Hwang and Lee 2003)
FAMILY CONIDAE	
*Conus flavidus* Lamarck, 1810	(Wu 2002, Hwang and Lee 2003)
*Conus lividus* Hwass, 1792	(Hwang and Lee 2003)
*Conus textile* Linnaeus, 1758	(Jung and Lai 1999, Wu 2002)
*Conus striatus* Linnaeus, 1758	(Wu 2002)
ORDER HETEROBRANCHIA	
FAMILY APLYSIIDAE	
*Aplysia juliana* Quoy & Gaimard, 1832	(Hwang and Lee 2003)
*Aplysia oculifera* Adams & Reeve, 1850	(Hwang and Lee 2003)
*Dolabrifera dolabrifera* (Rang, 1928)	(Hwang and Lee 2003)
FAMILY PHYLLIDIIDAE	
*Phyllidia pustulosa* Cuvier, 1804	(Hwang and Lee 2003)
*Phyllidia varicose* Lamarck, 1801	(Hwang and Lee 2003)
FAMILY SIPHONARIIDAE	
†*Siphonaria laciniosa* (Linnaeus, 1758)	§
FAMILY PLANORBIDAE	
†*Laevapex nipponica* (Kuroda, 1947)	§
FAMILY ELLOBIIDAE	
†*Carychium hachijoensis* Pilsbry, 1902	§
FAMILY VERONICELLIDAE	
Vaginulus alte (Ferussac, 1821)	§ (Wu 2002)
FAMILY SUCCINEIDAE	
†*Succinea erythrophana* Ancey, 1883	§
FAMILY CLAUSILIIDAE	
†*Zaptyx crassilamellata* Kuroda, 1941	§
FAMILY ACHATINIDAE	
*Achatina fulica* Bowdich, 1822	§ (Wu 2002)
FAMILY SUBULINIDAE	
†*Allopeas pyrgula* (Schmacker & Boettger, 1891)	§
FAMILY PHILOMYCIDAE	
*Meghimatium bilineatum* (Benson, 1842)	(Wu 2002)
FAMILY CAMAENIDAE	
*Coniglobus melleus* (Pfeiffer, 1865)	(Kuroda 1938, Kuroda 1941)
FAMILY BRADYBAENIDAE	
*Acusta despecta* (Sowerby, 1839)	(Kuroda 1938, Kuroda 1941)
*Aegista mackensii* (Adams & Reeve, 1850)	§ (Jung and Lai 1999, Wu 2002)
*Aegista osbeckii* (Philippi, 1847)	§ (Kuroda 1938, Kuroda 1941, Wu 2002)
*Bradybaena similaris* (Ferussac, 1822)	§ (Kuroda 1941)

†New records on Gueishan Island § Collected by our field sampling

### Taxonomic ranks

**Phylum:** Mollusca

**Class:** Bivalvia, Cephalopoda, Gastropoda, Polyplacophora

**Order:** Arcoida, Caenogastropoda, Heterobranchia, Neoloricata, Neritimorpha, Octopoda, Ostreoida, Patellogastropoda, Pterioida, Unionoida, Veneroida, Vetigastropoda

**Family:** Achatinidae, Aplysiidae, Arcidae, Argonautidae, Assimineidae, Bradybaenidae, Bursidae, Camaenidae, Cardiidae, Chitonidae, Clausiliidae, Columbellidae, Conidae, Corbiculidae, Cypraeidae, Diplommatinidae, Ellobiidae, Fasciolariidae, Haliotidae, Littorinidae, Lottiidae, Muricidae, Nassariidae, Neritidae, Octopodidae, Ostreidae, Ovulidae, Patellidae, Pectinidae, Philomycidae, Phyllidiidae, Planaxidae, Planorbidae, Potamididae, Pteriidae, Ranellidae, Siphonariidae, Subulinidae, Succineidae, Thiaridae, Trochidae, Turbinellidae, Turbinidae, Unionidae, Veronicellidae

**Genus:** *Achatina*, *Acusta*, *Aegista*, *Allopeas*, *Aplysia*, *Argonauta*, *Assiminea*, *Astralium*, *Barbatia*, *Batillaria*, *Bradybaena*, *Bursa*, *Calliostoma*, *Calpurnus*, *Carychium*, *Cellana*, *Chicoreus*, *Chlamys*, *Chlorostoma*, *Collisella*, *Coniglobus*, *Conus*, *Corbicula*, *Crassostrea*, *Cristaria*, *Cymatium*, *Cypraea*, *Diplommatina*, *Dolabrifera*, *Drupa*, *Ergalatax*, *Haliotis*, *Laevapex*, *Liolophura*, *Littoraria*, *Lottia*, *Lunella*, *Mancinella*, *Meghimatium*, *Monodonta*, *Morula*, *Nassarius*, *Nerita*, *Nodilittorina*, *Notoacmea*, *Octopus*, *Ovula*, *Patella*, *Peristernia*, *Phyllidia*, *Pinctada*, *Planaxis*, *Pteria*, *Purpura*, *Pyrene*, *Saccostrea*, *Siphonaria*, *Solenomphala*, *Stomatella*, *Succinea*, *Tarebia*, *Tectus*, *Telasco*, *Tenguella*, *Thais*, *Thiara*, *Tridacna*, *Trochus*, *Vaginulus*, *Vasum*, *Zaptyx*.

## Spatial coverage

**General spatial coverage:** The spatial coverage of the literature and our field investigation ranged from a latitude of 24°49'48"N to 24°51'0"N and a longitude of 121°55'48"E to 121°57'36"E. It includes the marine, intertidal, freshwater and terrestrial environment of Gueishan Island, Taiwan (Figure 1)

**Coordinates:** 24°49'48"N and 24°51'0"N Latitude; 121°55'48"E and 121°57'36"E Longitude

## Temporal coverage:

1934–2012.

## Methods

### Sampling description:

**Literature survey:** We searched for publications (including journals, project reports, theses and books) associated with the molluscan fauna of Gueishan Island from the following databases: (1) the National Digital Library of Theses and Dissertations in Taiwan (http://ndltd.ncl.edu.tw) (this contains details of theses and dissertations published since 1956, but did not contain publications relevant to this study); (2) the National Bibliographic Information Network (http://nbinet3.ncl.edu.tw) (this catalog integrates information from National Central Library and 74 other libraries containing all publications with a Taiwan ISBN and selected government project reports; three publications (Wu 2002, National Museum of Marine Biology and Aquarium 2003, Hwang and Lee 2003) from this database met our requirement); (3) the Government Research Bulletin (http://grbsearch.stpi.narl.org.tw/GRB/) (this contains government project reports made since 1993, but did not contain reports relevant to this study); (4) Google Scholar (http://scholar.google.com.tw/) (this contains a wide range of resources, from journals and books to webpages, and it provided two relevant journal articles (Chen and Fu 2007, Lee and Chen 2010)); (5) The Taiwan Malacofauna Database (http://shell.sinica.edu.tw/) (this database contains taxonomy, distribution and references of all mollusks occurred in Taiwan, and provided six relevant publications (Lee and Wu 1998, Jung and Lai 1999, Wu 2002, National Museum of Marine Biology and Aquarium 2003, Hwang and Lee 2003, Chen and Fu 2007)). In addition, three relevant publications (Hayasaka and Tan 1934, Kuroda 1938, Kuroda 1941) were identified from citations in Wu (2002). In total, we identified ten relevant publications. Three of these publications (Lee and Wu 1998, Chen and Fu 2007, Lee and Chen 2010) were excluded because they described specimens acquired from fishing ports that had been captured by shrimp fishing or bottom trawling boats near Gueishan Island, without information of the precise sampling location. The seven remaining publications were used to establish the occurrence and inventory data. Sampling sites, names of collectors and the scientific name of each species were recorded using Microsoft EXCEL 2010. All of the publications mentioned above can be accessed in the National Central Library and the National Taiwan Library.

**Field Sampling:** The topology of Gueishan Island and the types of mollusk habitat were considered for field investigation. Visual search was conducted for mollusks in intertidal, freshwater and terrestrial environments (Figure 1). The surface of rocks on the coastline and man-made concrete structures in port were searched for marine mollusks during low tide. Leaf litter and rocks under or near water around Tail Lake (the only freshwater habitat on island) were inspected for freshwater mollusks. We inspected from leaves, trunks, leaves litter, rocks and rotten woods for land snails along three trails: one trail around Tail Lake, another leads to the highest peak (401 Highland) on the island, and the other leads to the northern part of the island. We surveyed for land snails during their active periods: during and after rainfall, early morning, and night. At least one living individual or dead shells of each species was collected as voucher specimens. Living organisms were brought back to laboratory, fixed via freezing in a -80°C freezer, and subsequently transferred to 95% ethanol for long term preservation.

**Quality control description:** Latitude, longitude and altitude of sampling sites were recorded using Garmin *GPSmap 60CSx* with uncertainty of less than 10 meters. Sampling sites were georeferenced (WGS84). All the specimens collected during the field investigation were identified independently by Huang and Hsiung. Seven earlier studies described the mollucan fauna of Gueishan Island, but these publications lack clear photos or other information for identifying specimens. Species identification was performed using the following guide books and publications about Taiwan malacofauna: Pace (1973), Lai (1990, 1998), Lee and Chen (2003), Wu and Lee (2005), and Hsieh et al. (2006). Newly recorded species were further confirmed by Dr. Yen-Chen Lee, a Mollusca specialist and postdoctoral researcher in the Biodiversity Research Center, Academia Sinica. Fourteen new recorded species were found to be native to Taiwan but previously unreported on Gueishan Island. The scientific names of all mollusks were checked against the Taiwan Malacofauna Database and World Register of Marine Species (http://www.marinespecies.org/).

## Data resources

The data underpinning the analysis reported in this paper are deposited at GBIF, the Global Biodiversity Information Facility, http://taibif.org.tw/ipt/resource.do?r=gueishan_island

## Datasets

**Dataset description:** This dataset incorporates seven publications (Hayasaka and Tan 1934, Kuroda 1938, Kuroda 1941, Jung and Lai 1999, Wu 2002, National Museum of Marine Biology and Aquarium 2003, Hwang and Lee 2003) associated with the molluscan fauna of Gueishan Island and field investigation results. The dataset includes sampling date, taxonomy information, GPS location, elevation, type of habitat, name of collector, method of collection, and literature record. Based on the literature published during the period between 1934 and 2003, 112 species from 61 genera and 37 families of Mollusca were recorded on Gueishan Island. Of the 34 species from 28 genera and 23 families identified during our 2011-2012 field investigation, fourteen species were new records on Gueishan Island. In total, our literature survey and field investigation documents 126 species from 71 genera and 45 families of Mollusca on Gueishan Island. The fourteen newly recorded species are: *Liolophura japonica* (Lischke, 1873), *Lottia luchuana* (Pilsbry, 1901), *Siphonaria laciniosa* (Linnaeus, 1758), *Nerita costata* Gmelin, 1791, *Nerita rumphii* Recluz,1841, and *Littoraria undulata* (Gray, 1839), which were sampled from the marine environment; *Assiminea* sp. and *Laevapex nipponica* (Kuroda, 1947), which were discovered in a freshwater environment, Tail Lake; and *Solenomphala taiwanensis* (Habe, 1942), *Diplommatina suganikeiensis* (Pilsbry & Hirase, 1905), *Carychium hachijoensis* Pilsbry, 1902, *Zaptyx crassilamellata* Kuroda, 1941, *Allopeas pyrgula* (Schmacker & Boettger, 1891), and *Succinea erythrophana* Ancey, 1883 discovered in the terrestrial environment. This dataset provide basic information for the island’s biodiversity and biogeography. This dataset will be maintained by the Malacology Lab, Biodiversity Research Center, Academia Sinica.

**Object name:** Darwin Core Archive Molluscan fauna of Gueishan Island, Taiwan

**Character encoding:** UTF-8

**Format name:** Darwin Core Archive format

**Format version:** 1.0

**Distribution:** http://taibif.org.tw/ipt/archive.do?r=gueishan_island

**Publication date of data:** 2012-12-21

**Language:** English

**Licenses of use:** This work is licensed under a Creative Commons CCZero 1.0 License http://creativecommons.org/publicdomain/zero/1.0/legalcode

**Metadata language:** English

**Date of metadata creation:** 2012-09-21

**Hierarchy level:** Dataset
